# 2026 Asia Pacific Heart Rhythm Society (APHRS) Guidelines (Part I) on Epidemiology, Detection/Screening and Integrated Patient Care in Atrial Fibrillation

**DOI:** 10.1002/joa3.70495

**Published:** 2026-10-21

**Authors:** Boyoung Joung, Rungroj Krittayaphong, Yutao Guo, Pil‐Sung Yang, Yu‐Cheng Hsieh, Takanori Ikeda, Daehoon Kim, Pipin Kojodjojo, Ngai‐Yin Chan, Yoga Yuniadi, Kieu Ngoc Dung, Sirin Apiyasawat, Wael Almahmeed, Eue‐Keun Choi, Melissa E. Middeldorp, Kyoko Soejima, Gregory Y. H. Lip, Hui‐Nam Pak, Tze‐Fan Chao

**Affiliations:** ^1^ Division of Cardiology, Department of Medicine Severance Hospital, Yonsei University College of Medicine Seoul Korea; ^2^ Division of Cardiology, Department of Medicine Siriraj Hospital, Mahidol University Bangkok Thailand; ^3^ Department of Pulmonary Vessel and Thrombotic Disease, Sixth Medical Center Chinese PLA General Hospital Beijing China; ^4^ Division of Cardiology, Department of Medicine Yongin Severance Hospital, Yonsei University College of Medicine Yongin Korea; ^5^ Cardiovascular Center Taichung Veterans General Hospital, National Chung Hsing University Taichung Taiwan; ^6^ Division of Cardiovascular Medicine, Department of Internal Medicine Toho University Faculty of Medicine Tokyo Japan; ^7^ Asian Heart and Vascular Centre Singapore Singapore; ^8^ Department of Medicine and Geriatrics Princess Margaret Hospital Hong Kong China; ^9^ Department of Cardiology and Vascular Medicine Universitas Indonesia Jakarta Indonesia; ^10^ Cho Ray Hospital Ho Chi Minh Vietnam; ^11^ Division of Cardiology, Department of Medicine Ramathibodi Hospital, Mahidol University Bangkok Thailand; ^12^ Heart and Vascular Institute Cleveland Clinic Abu Dhabi UAE; ^13^ Department of Internal Medicine Seoul National University Hospital, Seoul National University College of Medicine Seoul Republic of Korea; ^14^ Centre for Heart Rhythm Disorders Adelaide University Adelaide South Australia Australia; ^15^ Department of Cardiology Kyorin University School of Medicine Tokyo Japan; ^16^ Institute of Life Course and Medical Sciences University of Liverpool Liverpool UK; ^17^ Department of Clinical Medicine Aalborg University Aalborg Denmark; ^18^ Department of Cardiology, Lipidology and Internal Medicine With Intensive Coronary Care Unit Medical University of Bialystok Bialystok Poland; ^19^ Cardiovascular Center Taipei Veterans General Hospital Taipei Taiwan

## Abstract

The 2026 Asia Pacific Heart Rhythm Society (APHRS) Guidelines represent the first comprehensive APHRS guidelines dedicated to the management of atrial fibrillation (AF) and comprise four parts covering epidemiology, detection/screening and integrated care; stroke prevention; rate and rhythm control; and lifestyle risk factor and comorbidity management. Part 1 focuses on epidemiology and integrated care management, integrating contemporary global evidence with data and clinical considerations specific to the Asia‐Pacific region. It provides evidence‐based and practical recommendations for clinical epidemiology in the region, as well as introduces the need for holistic or integrated care management.

## 1 Epidemiology

Atrial fibrillation (AF) is the most prevalent sustained cardiac arrhythmia worldwide. Over the past two decades, both the incidence and prevalence of AF have risen substantially and are projected to continue increasing over the next 30 years, particularly in countries with a middle sociodemographic index. AF represents a growing global public health challenge [1].

### 1.1 Prevalence and Incidence of AF

The recently reported community‐based prevalence of AF in Asia‐Pacific Region ranges from 1.07% to 1.92% (1.92% in South Korea, 1.07% in Taiwan, 1.87% in China, 1.4% in Japan, 1.41% in Australia, 1.7% in New Zealand, 1.6% in India, and 1.5% in Singapore [2, 3, 4, 5, 6, 7, 8, 9]).

The prevalence of AF in hospital‐based settings is typically higher than in community settings, as these populations include patients with more comorbidities. In South East, Central, and Western Asia, most data on AF prevalence and incidence have been derived from hospital‐based populations rather than community cohorts. A comprehensive meta‐analysis including 58 studies conducted across Asia demonstrated substantial variation in AF prevalence in hospital populations, ranging from 2.8% to 15.8% (3.2% in Indonesia, 3.3% in Kazakhstan, and 6.6% in Israel) (Table 1) (Figure 1) [18, 19, 20, 21, 22, 24, 25, 26, 27]. The prevalence of AF has progressively increased in most Asia‐Pacific countries: in Korea, from 0.73% in 2006 to 1.92% in 2021; in Taiwan, from 0.46% in 2000 to 1.07% in 2011; and in Japan, from 0.8% in 2008 to 1.4% in 2015 [2, 3, 5, 10]. The prevalence of AF in Korea is projected to reach 5.81% by 2060 [19], while in China and Taiwan it is estimated to reach 3.70% and 4.01%, respectively, by 2050 (Figure 2) [3]. AF prevalence is consistently higher in men than in women [2, 3, 5, 10]. Across all age groups, prevalence has increased over recent decades, except among individuals aged 20–29 years, in whom a significant decrease has been observed (Figure 3A) [3, 10].

**TABLE 1:** Prevalence and incidence of AF in the Asia‐Pacific and Australia.

Country	Study year(s)	Sample size (*n*)	Data source	Age (years)	Prevalence (%) (men, women)	Incidence (per 1000 person‐year) (men, women)	References
East Asia							
South Korea	2006–2015	679 416	Korean National Health Insurance Service database	≥ 20	0.73 (0.77, 0.69)	1.83 (1.91, 1.75)	Kim et al. (2018) [10]
					1.53 (1.63, 1.43)	1.77 (1.89, 1.65)	
South Korea	2021	1 185 366	Korean National Health Insurance Service database	≥ 20	1.92 (2.13, 1.72)	2.69 (2.95, 2.44)	Kim et al. (2025) [2]
South Korea	2022	1 185 366	Korean National Health Insurance Service database	≥ 20	2.2 (2.4, 2.0)	2.75 (3.01, 2.49)	Lee et al. (2025) [11]
Taiwan	2000	289 559	Taiwan National Health Insurance Research Database	≥ 20	0.46	1.51	Chao et al. (2018) [3]
	2011				1.07	1.51	
China	2014–2015	726 451	China National Stroke Screening and Prevention Project	≥ 60	2.31 (2.72, 1.90)		Wang et al. (2018) [12]
China	2020–2021	114 039	A national cross‐sectional epidemiological study	≥ 18	1.6		Shi, et al.(2022) [13]
China	2015–2019	18 738	Community‐based data in Shanghai, China	≥ 60	1.87	5.2	Wei, et al.(2023) [4]
Japan^a^	2008	630 137	Tama City database excluding paroxysmal AF	≥ 40 and < 75	0.8 (1.7, 0.2)	2.5 (4.5, 1.3)	Kodani et al. (2019) [5]
	2015				1.4 (2.9, 0.4)		
Japan^a^	2003	630 137	Examinations from company clinics, local governments and health centers excluding paroxysmal AF	≥ 40	0.86 (1.35, 0.43)		Inoue et al. (2019) [14]
Hong Kong	2014–2015	13 122	A territory‐wide cross‐sectional screening and epidemiological study	≥ 18	1.8 (2.8, 1.4)		Chan et al. (2017) [15]
Australia and New Zealand							
Australia	2001–2003	1035	Crossroads I study	≥ 18	1.6		Glenister et al. (2023) [16]
	2016–2018	715	Crossroads II study		1.8		
Australia	1999–2000	8273	National, population‐based survey	≥ 35	1.41 (1.46, 1.36)	2.0 (2.8, 1.3)	Diouf et al. (2016) [6]
New Zealand	2014	739 000	Electronic medical records from 170 general practices	All	1.7 (2.1, 1.4)		Tomlin et al. (2017) [7]
South Asia							
India	Unknown	4077	Community‐based survey in Nagpur	≥ 18	0.19 (0.1, 0.3)		Saggu et al. (2016) [17]
India	2015	2100	SMART‐India study (rural region of Anand district, Gujarat, India)	≥ 40	1.6 (2.3, 1.0)		Soni et al. (2018) [8]
South East Asia							
Thailand	Unknown	1277	Primary health care centre survey in Chiang Mai	≥ 65	1.9 (2.0, 1.8)		Phrommintikul et al. (2016) [18]
Thailand	2018	13 864	Local primary care nurses and village health volunteers in Phetchaburi and Lopburi provinces	≥ 65	2.8		Suwanwela et al.(2021) [19]
Malaysia	2007–2014	10 805	Population‐based study in various urban and rural communities	≥ 30	0.5 (0.7, 0.4)		Lim et al. (2016) [20]
Malaysia	2011	1998	Outpatient hypertension clinic	≥ 30	0.8 (0.8, 0.8)		Wong et al. (2013) [21]
Singapore	2004–2008	1839	Community‐based screening project	≥ 55	1.5 (2.6, 0.6)		Yap et al. (2008) [9]
Indonesia^b^	2018–19	9773	Referral hospitals visitors		3.2		Yuniadi et al. (2024) [22]
Vietnam	2013	—	—	—	1.0	—	Lien et al. (2013) [23]
Central Asia							
Central Asia	1990–2021		Global Burden of Disease 2021		0.54	0.45	Cheng et al. (2024) [24]
Kazakhstan	2015	1575	Cross‐sectional population‐based survey	18–69	3.30		Akpanova et al.(2019) [25]
Western Asia							
Western Asia	1990–2021		Global Burden of Disease 2021		0.37	0.35	Cheng et al. (2024) [24]
Israel	2012–2014	1 137 198	Clalit Health Service Database	≥ 50	6.6	7.6	Saliba et al.(2016) [26]

^a^ The reported prevalence of atrial fibrillation (AF) in Japan was calculated after excluding paroxysmal AF. Therefore, the true prevalence of AF in Japan is likely to be approximately twice the currently reported figure.

^b^ The higher reported AF prevalence likely reflects tertiary hospital‐based data, as national population‐based estimates are lacking.

**FIGURE 1:**
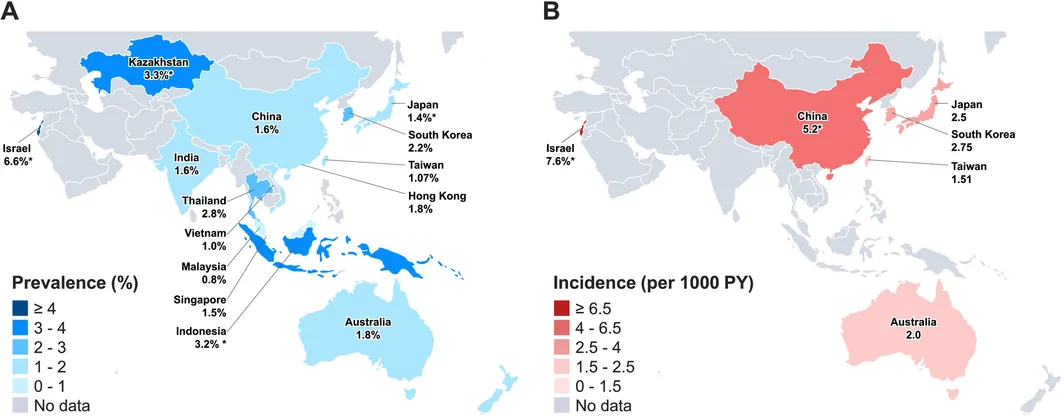
Prevalence (A) and incidence (B) of AF in the Asia‐Pacific and Australia. *The reported prevalence of AF in Japan was calculated after excluding paroxysmal AF. Therefore, the true prevalence of AF in Japan is likely to be approximately twice the currently reported figure. The higher reported AF prevalence of Indonesia, Kazakhstan, and Israel likely reflects tertiary hospital‐based data, as national population‐based estimates are lacking. The higher reported AF incidence of China and Israel likely reflects tertiary hospital‐based data, as national population‐based estimates are lacking.

**FIGURE 2:**
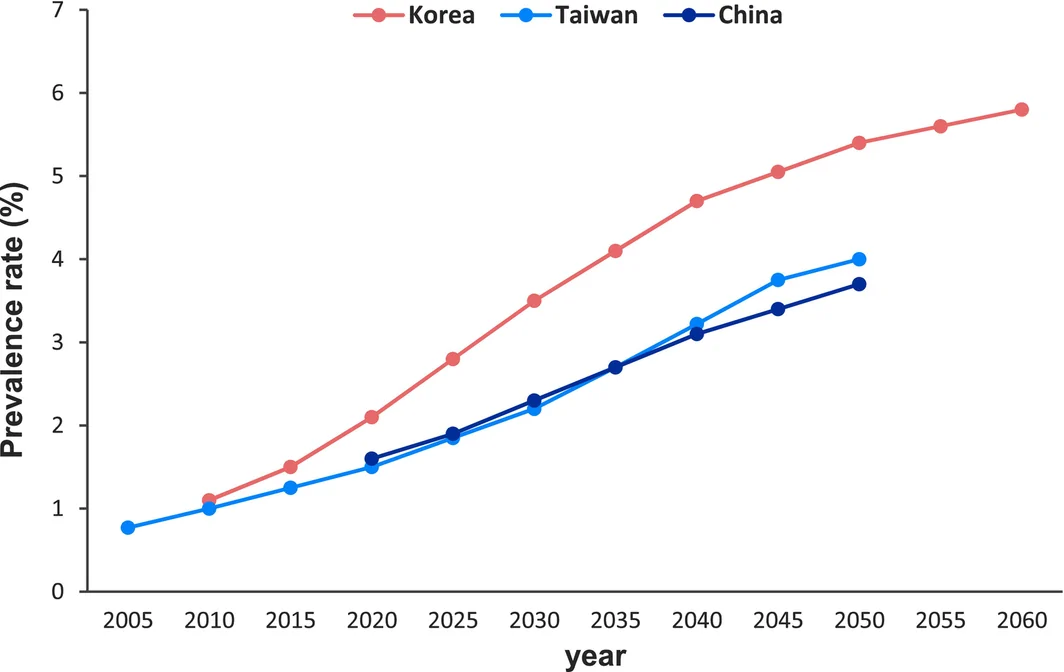
Projected prevalence rate of AF in Korea, Taiwan and China.

**FIGURE 3:**
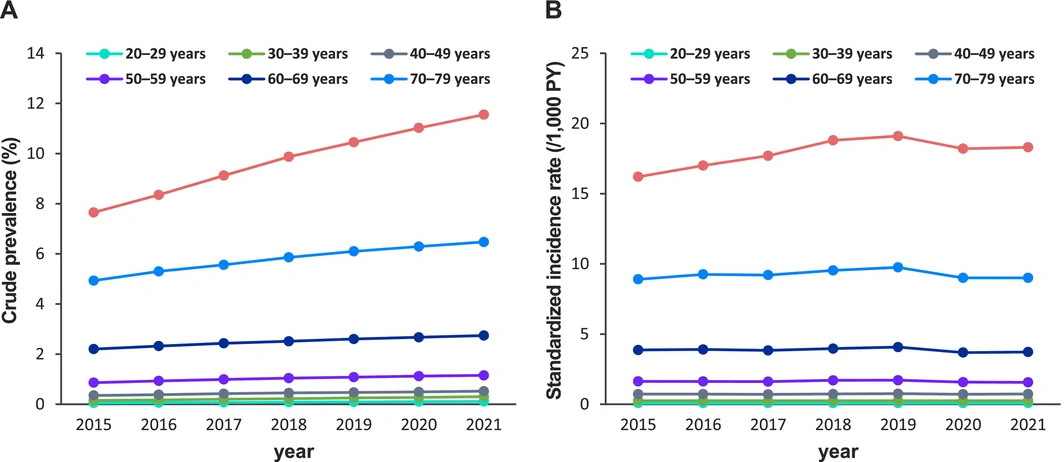
(A) Annual prevalence of atrial fibrillation from 2015 to 2021 in Korea; *p* for trend < 0.01 in all age groups. (B) Annual age‐ and sex‐standardized incidence of atrial fibrillation from 2015 to 2021; stratified by age group in Korea. *p* for trend < 0.01 in all age groups except for those in their 60s.

The incidence of AF in community‐based populations in Asia ranges from 1.51% to 2.69% (2.69% in Korea, 1.51% in Taiwan, 2.5% in Japan, and 2.0% in Australia [6]) (Table 1) [2, 3, 5, 6]. The overall incidence is higher in men than in women, a trend that has remained consistent over the past decade [2]. Notably, the annual incidence of AF has increased significantly among individuals aged ≥ 80 years, whereas incidence rates in other age groups have remained relatively stable (Figure 3B) [2].

### 1.2 Prognosis of AF

Among Asian patients with AF, the annual risk of ischemic stroke is approximately 3.0% (range, 1.60%–4.95%) based on pooled analyses of eight studies [27]. In non‐anticoagulated AF cohorts from Korea and Taiwan, the annual risk of ischemic stroke was 3.3% and 3.4%, respectively—approximately threefold higher than in individuals without AF [3, 28]. Compared with patients diagnosed with AF in 2015 in Korea, those diagnosed in 2019 had an 11% lower risk of ischemic stroke. Furthermore, compared with patients with incident AF diagnosed in 2011, there was a progressively greater reduction in ischemic stroke risk during 2012–2020, while the risk of intracranial hemorrhage did not change significantly [2]. Standardized mortality ratios, defined as the ratio of observed deaths among patients with AF to expected deaths in the general population, declined over time in many Asian countries [2, 3]. Temporal trends in 1‐year event rates decreased in patients with AF (Figure 4A). The risks of adverse events including mortality, heart failure, ischemic stroke, and myocardial infarction were increased by AF (Figure 4B) [3, 11].

**FIGURE 4:**
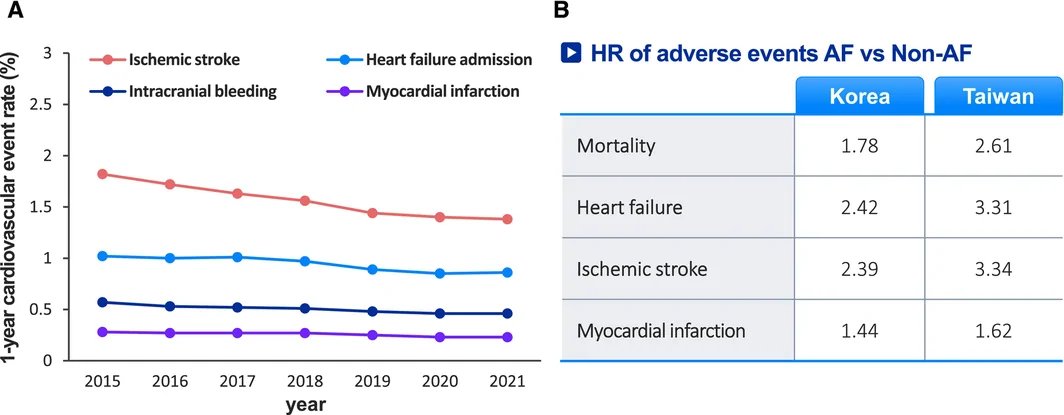
(A) Temporal trends in 1‐year event rates of patients with atrial fibrillation. (B) Risk of adverse events including mortality, heart failure, ischemic stroke, and myocardial infarction by atrial fibrillation.

The observed reduction in stroke risk may be attributed to increased initiation of direct oral anticoagulants (DOACs) in newly diagnosed patients with AF [29]. In Korea, DOAC use increased significantly from 44.6% in 2013 to 77.5% in 2022 [11]. Similarly, in Taiwan, DOAC prescription rates rose from 13.6% in 2012 to nearly 60.0% by 2020 [30]. Increased use of oral anticoagulants (OACs), particularly following the introduction of DOACs, was associated with a reduction in ischemic stroke‐related emergency department visits without a significant increase in major bleeding events [31]. Although the increasing use of OACs—particularly DOACs—has led to substantial reductions in stroke and cardiovascular events, a more integrated approach to AF management is needed to effectively address the overall healthcare burden and associated risks.

The impact of AF on healthcare costs reflects its association with increased morbidity and mortality, including stroke, heart failure, and hospitalizations, and is projected to rise further in Asia over the coming decades [32]. Data from the UK suggest that AF accounts for approximately 0.9%–1.6% of National Health Service (NHS) expenditure, largely driven by hospital admissions [33]. In Korea, the total cost of AF care accounted for approximately 0.78% of National Health Insurance Service (NHIS) expenditure in 2015 [34].

The risks of stroke and mortality in AF are closely associated with multiple cardiovascular and non‐cardiovascular comorbidities, which often cluster as multimorbidity and adversely affect prognosis [35]. The clinical characteristics of patients with AF have evolved over time, reflecting increased risk associated with population aging. In Korea, the proportion of patients aged ≥ 75 years increased from 34.9% in 2017 to 43.9% in 2022 [2, 11], while the proportion of patients with a CHA_2_DS_2_‐VASc score ≥ 2 rose from 74.6% in 2015 to 79.5% in 2021 [2, 10]. The prevalence of comorbid conditions—including hypertension, diabetes mellitus, heart failure, and ischemic stroke—has also increased. Similarly, the proportion of patients with a high bleeding risk (HAS‐BLED score ≥ 3) has risen [10]. In Taiwan, the mean CHA_2_DS_2_‐VASc score increased steadily from 3.53 in 2000 to 4.44 in 2011 [3].

## 2 AF Detection/Screening

### 2.1 Recent Evidence for AF Screening

AF screening is undergoing a significant paradigm shift, driven by the rapid integration of digital health technologies. AF screening strategies range from traditional, clinic‐based opportunistic screening to technology‐facilitated, systematic, and consumer‐led approaches. Targeted screening is recommended for individuals aged ≥ 75 years, as well as for older adults aged ≥ 65 years with additional CHA_2_DS_2_‐VA risk factors or obstructive sleep apnea (OSA) to ensure earlier detection of AF [36, 37]. Consumer‐led screening empowers individuals to use validated wearable devices (e.g., smartwatches with photoplethysmography (PPG) or single‐lead ECG) for self‐monitoring for AF, which democratizes screening, enabling large‐scale, convenient, and continuous monitoring outside clinical settings [38, 39].

The optimal approach of AF screening depends on the target population and available resources, as more sensitive monitoring methods improve AF detection but may increase false positives and identification of low‐burden AF. Trials evaluating single timepoint screening have generally shown limited effectiveness and no randomized evidence currently demonstrates improved clinical outcomes from such strategies [40, 41]. Conversely, studies using prolonged screening strategies have consistently shown an increase in AF detection and the initiation of OAC [41, 42]. This aligns with the recent AMALFI trial, which demonstrated that a remote, 14‐day continuous ECG patch in older adults at moderate‐to‐high stroke risk led to a modest but significant increase in long‐term AF detection (6.8% vs. 5.4%) and greater OAC prescription compared to usual care [43].

Despite the proven ability to increase detection, the impact of prolonged screening on major clinical outcomes remains uncertain. While the STROKESTOP trial (using intermittent single‐lead ECGs) showed a marginal reduction in a composite outcome including stroke and mortality [44], the LOOP trial (using implantable loop recorders) found no reduction in stroke risk [45]. Using a biomarker‐guided approach (NT‐proBNP) to target prolonged intermittent ECG screening in 75‐ and 76‐year‐olds, the STROKESTOP II trial found that this strategy neither increased overall AF detection nor prevented stroke and systemic embolism compared to usual care [46]. Similarly, the *AMALFI* trial reported no significant difference in stroke events, noting that over half of the newly detected AF was low‐burden [43].

Overall, while prolonged and digitally enhanced monitoring effectively identifies previously undiagnosed AF, its ability to significantly reduce hard clinical endpoints remains unproven. Further large‐scale, long‐term studies are needed to clarify the true clinical benefit and cost‐effectiveness of these screening strategies.

### 2.2 Screening Tools for AF

With the advancement in technology, various tools including consumer wearables have become widely available for AF screening (Table 2) [63]. Single‐lead ECG reviewed by a physician is acceptable in the diagnosis of AF during screening [64]. Therefore ECG devices for AF screening are more preferable than non‐ECG tools since a confirmatory ECG is required for the latter. Single‐lead or multiple‐lead ECG's can be performed by handheld or wearable devices. In the STROKESTOP study, the Zenicor handheld device similarly producing a lead I ECG was used. The automatic diagnostic algorithm achieved a sensitivity of 98% and specificity of 88% in diagnosing AF [50].

**TABLE 2:** Diagnostic accuracy of different tools for atrial fibrillation screening.

Screening methods/tools	Authors	Sensitivity (%)	Specificity (%)	PPV (%)	NPV (%)
Pulse palpitation	Taggar et al. [47]	92 (85–96)	82 (76–88)	N/A	N/A
	Cooke et al. [48]	94 (84–97)	72 (69–75)	N/A	N/A
Handheld or wearable ECG devices					
AliveCor (Kardia) Heart Monitor	Chan NY et al. [49]	75 (70–80)	98 (98–98.4)	65 (59–71)	99.5 (99.4–99.6)
Zenicor II	Svennberg et al. [50]	98 (96–100)	88.2 (88–88.4)	2.8 (2.5–3.1)	100
Mydiagnostick	Tieleman et al. [51]	100 (93–100)	96 (91–98)	90 (82–98)	100
	Vaes et al. [52]	94 (87–98)	93 (85–97)	94 (89–99)	93 (88–98)
Omron HCG‐801 Monitor	Kearley et al. [53]	99 (93–100)	76 (73–79)	26 (21–32)	99.9 (99–100)
Apple Watch AliveCor Kardia Band	Bumgarner et al. [54]	93 (86–98)	84 (73–95)	90 (83–97)	88 (78–98)
Modified blood pressure monitors					
Microlife WatchBP Home A	Chan PH et al. [55]	83 (68–98)	99 (98–99)	43 (29–57)	99.8 (99.6–100)
Microlife BPA 200 plus	Marazzi et al. [56]	92 (87–97)	95 (93–97)	83 (76–90)	98 (97–99)
Omron M6	Marazzi et al. [56]	100	94 (92–97)	82 (75–88)	100
Omron M6 Comfort	Wiesel et al. [57]	30 (15–49)	97 (93–99)	69 (44–94)	88 (83–93)
Photo‐plethysmographic devices					
iPhone photo‐plethysmography	McManus et al. [58]	97 (94–100)	94 (89–98)	92 (87–97)	97 (95–100)
Cardiio Rhythm	Chan PH et al. [59]	93 (77–99)	98 (97–99)	53 (38–67)	99.8 (99–100)
Cardiio Rhythm facial photo‐plethysmography	Yan et al. [60]	95 (87–98)	96 (91–98)	92 (84–96)	97 (93–99)
Samsung Gear Fit 2 smartwatch (1‐min pulse plethysmography analysis)	Dörr et al. [61]	94 (90–96)	98 (96–99)	98 (95–99)	95 (91–97)
Huawei wristwatch	Fan et al. [62]	95 (92–97)	99 (98–100)	99 (98–100)	96 (93–98)

*Note:* Numbers in parentheses represent 95% confidence intervals.

Abbreviations: ECG, electrocardiogram; N/A, not applicable; NPV, negative predictive value; PPV, positive predictive value.

Modified blood pressure monitors have also been used for AF screening [55]. In this device, an irregularity index represented by the ratio of the standard deviation of successive R‐R intervals to the mean R–R intervals is calculated and AF is diagnosed when a certain cut‐off threshold is exceeded.

PPG waveform analysis has been incorporated into smartphone and smartwatch platforms for AF screening. A smartphone camera‐based pulse PPG waveform measurement algorithm from Cardiio Rhythm for detecting AF was studied in 1013 patients with hypertension, diabetes and/or aged ≥ 65 years in a primary healthcare setting [59]. PPG waveforms were acquired when the finger of an individual was placed over the camera of the smartphone and illuminated by the LED flash. The reflected light captured by the camera changes according to the pulsatile arterial blood volume and AF is diagnosed by a lack of repeating pattern in the PPG waveforms. This diagnostic algorithm was found to have a sensitivity of 93% and specificity of 98% in detecting AF. Facial pulsatile PPG signals from Cardiio Rhythm were tested for contact‐free AF screening in 217 patients with a sensitivity of 95% and specificity of 96% in AF detection [60].

The AliveCor ECG technology incorporated in the Apple Watch was studied in patients before and after AF cardioversion [54]. Notably, 34% of ECG's were classified as uninterpretable by the algorithm. After exclusion of the uninterpretable ECG's, the sensitivity and specificity of the AF diagnostic algorithm were 93% and 84%, respectively.

### 2.3 AF Screening in Asia

AF detection rates are closely linked to both technological advances in screening methods and the screening strategies employed. During a Japanese community‐based screening of 1607 elderly participants (mean age 72.4 years) using a combined blood pressure monitor and ECG device, AF was newly identified in 15 individuals, representing a detection rate of only 0.93% [65]. If such screening exercises were repeated annually over 4 years for subjects above 65 years old, a new AF yield of 0.33% to 0.55% per year could be achieved [66]. In an AF screening study in Hong Kong, a modified blood pressure monitor (Microlife WatchBP Home A) showed a sensitivity and specificity of 83% and 99% [55].

Sun et al. investigated AF screening with opportunistic handheld ECG and stroke risk in a Chinese cohort aged 55 to 64 years. Whilst AF detection yield was modest at 0.8%–1.0%, the identified patients had an increased stroke risk, with most of those with at least 1 risk factor independent of age and gender [67]. In a community‐based AF screening program in Hong Kong, the AliveCor (Kardia) Heart Monitor producing a lead I ECG showed a specificity of 98% and sensitivity of 75% [49].

Based on a worldwide systematic review of AF cases identified by single time point screening, the number needed to screen for 1 treatable new AF case is 83 for those 65 years or older and 1089 for those below 60 years of age [68]. Thus there is a strong age dependence for the number needed to screen using single time point ECG screening, with significant cost‐effectiveness implications when relying on such a strategy. According to a Markov decision‐making model, one‐time population screening for AF using a single lead handheld device in a cohort of 10 000 patients aged 75 years in Taiwan would prevent 45 ischemic strokes and gain 47.4 QALYs, resulting in an incremental cost‐effectiveness ratio of $12 493 per QALY gained [69]. This suggests that targeted systematic screening of AF in a higher risk Asian sub‐group can be cost effective.

Data from large‐scale, consumer‐led screening in China, which utilized Huawei wristwatches (e.g., Huawei Watch GT) with PPG technology for continuous pulse rhythm monitoring, showed that the prevalence of detected AF in a population averaging 35 years of age rose from about 0.23% in 2019 to 0.43% in 2022 [38, 39]. The detection rate rose progressively from 0.10% within the first 14 days to 0.83% by month 12, and further to 1.58% by the third year [39]. Such prolonged, continuous monitoring was essential for capturing paroxysmal AF, which was often missed by short duration or opportunistic checks. Whilst consumer technology screening of AF is advantageous in its unparalleled large scale and relatively low cost to healthcare providers, integration with local health services to provide AF management is essential. To date, this translation from better AF detection using consumer technology to improved clinical outcomes such as reduced stroke incidence has not been demonstrated.

Combining AI and big data analytics with wearable technology could enhance AF monitoring [70, 71, 72, 73]. A sequential two‐stage AI ECG screening protocol for AF has been shown to maintain a high negative predictive value at initial screening and improve the positive predictive value through longitudinal confirmation [74]. Once validated for clinical use, such an approach could improve diagnostic accuracy, direct monitoring resources to patients most likely to benefit, and enhance the cost‐effectiveness of screening. Furthermore, leveraging smart technologies, including wearables and AI, enables real‐time detection of AF episodes, facilitating the continuous tracking of AF dynamics and burden evolution. A five‐dimensional AF burden model, encompassing episode frequency, duration, temporal clustering, circadian patterns, and tachycardia burden, has been developed to capture the full spectrum of AF progression, linked to the clinical outcomes [75, 76]. This multidimensional approach promises to shift the paradigm from traditional symptom‐driven, retrospective management toward a proactive, progression‐based treatment strategy.

**Recommendation Table**.

**Table:** 

Recommendation	Class	Level
Targeted screening is recommended for individuals aged ≥ 75 years, as well as for older adults aged ≥ 65 years with additional CHA_2_DS_2_‐VA risk factors or obstructive sleep apnea (OSA) to ensure earlier detection of AF [36, 37, 68]	I	A
Consumer‐led screening using validated wearable devices (e.g., smartwatches with PPG or ECG) is considered as an effective strategy for the large‐scale detection of AF, enabling both the identification of AF and the concurrent assessment of associated risk factors [38, 39]	IIa	B
The integration of AI and wearable technology could enhance the diagnostic accuracy of AF screening, and longitudinal monitoring of AF burden and progression [74, 75, 76]	IIa	C

### 2.4 Device‐Based and CIED Screening for AF

Device‐based screening has become one of the most important frontiers in AF detection, particularly through cardiac implantable electronic devices (CIEDs) such as pacemakers, ICDs, and CRT devices. These devices allow continuous atrial rhythm monitoring, enabling detection of atrial high‐rate episodes (AHRE), a form of subclinical AF distinct from clinically diagnosed AF on surface ECG, as formally defined in the 2017 EHRA/HRS/APHRS/SOLEACE consensus document [77]. Early studies established that AHRE is associated with an increased stroke risk, though this risk is lower than that seen in clinical AF [78, 79]. The pivotal question of whether to anticoagulate these patients was directly addressed by two randomized trials, NOAH‐AFNET 6 [80] and ARTESiA [81]. A combined meta‐analysis of these two trials showed that oral anticoagulation with edoxaban or apixaban reduced the relative risk of stroke by approximately 32% while increasing the relative risk of major bleeding by approximately 62%. Because absolute stroke rates in device‐detected AF were low (1.1%–1.2% per year without anticoagulation), the absolute benefit of anticoagulation was modest, preventing roughly 0.3 strokes per 100 patient‐years while causing 0.7 to 1.6 additional major bleeding events per 100 patient‐years [82]. These findings underscore that decisions on anticoagulation in device‐detected AF require individualized risk stratification incorporating AHRE burden and CHA_2_DS_2_‐VASc score, rather than a uniform treatment approach. Implantable loop recorders (ILRs) have similarly proven valuable for AF detection, especially in cryptogenic stroke populations, as shown in the LOOP study [45]. Looking forward, continuous CIED‐ and wearable‐based monitoring is expected to play an increasingly central role in AF screening and recurrence surveillance. In 234 Indian patients with CIEDs, AHREs were recorded in 48 (21%) patients [83]. In a total of 816 consecutive Korean patients with CIEDs, the use of machine learning‐based models in patient classification was associated with improved prediction of clinically relevant AHREs after CIED implantation [84].

### 2.5 AF Prediction in Asia

AF risk stratification scores derived and validated in Asian populations provide important context for regional screening strategies. Recently, 2 schemes have been developed for the prediction of incident AF for patients in Asia and Taiwan, namely, the C_2_HEST (coronary artery disease or chronic obstructive pulmonary disease [1 point each]; hypertension [1 point]; elderly [age more than or equal 75 years, 2 points]; systolic heart failure [2 points]; thyroid disease [hyperthyroidism], 1 point) score and Taiwan AF score, respectively [85, 86]. The C_2_HEST score, originally derived from 471 446 Chinese subjects and externally validated in 451 199 Korean subjects, demonstrated superior discrimination for incident AF compared with CHADS_2_ and CHA_2_DS_2_‐VASc in both cohorts [85]. Taiwan AF score was derived from 7 220 654 Taiwan patients with 438 930 incident AF cases [85, 86]. The area under the curve for the prediction of AF was 0.749 (95% CI: 0.729–0.769) for the simple C_2_HEST score and 0.756 (95% CI: 0.755–0.757) for the Taiwan AF score [85, 86].

## 3 Integrated Patient Care

### 3.1 Patient‐Centered, Multidisciplinary AF Management

AF extends beyond an isolated electrophysiological disorder; it represents a complex clinical syndrome. In many cases, AF constitutes the final common pathway for multiple cardiac and non‐cardiac comorbidities, underscoring the need for a comprehensive, integrated care approach [87]. Evidence consistently demonstrates that an integrated care approach reduces cardiovascular hospitalization rates and decreases all‐cause mortality [88, 89].

This integrated strategy must address multiple therapeutic domains, including stroke prevention, rate or rhythm control, optimization of cardiovascular comorbidities, and lifestyle interventions. Critically, this approach must be inherently patient‐centered, with therapeutic strategies tailored to each patient's unique clinical circumstances, preferences, and values. Given the chronic and progressive nature of AF, maintaining continuity of care is crucial for achieving long‐term success [89, 90, 91].

To accomplish this complex task, a multidisciplinary approach with a dedicated framework is required. The framework must be sufficiently pragmatic to be broadly applicable across diverse patient populations and implementable by a wide range of healthcare personnel. As previously endorsed in the 2021 APHRS Consensus Guidelines, we continue to recommend the “ABC” (Atrial fibrillation Better Care) pathway [92], a streamlined, evidence‐based framework for integrated AF care (Figure 5). This strategy is defined by three main components:

**FIGURE 5:**
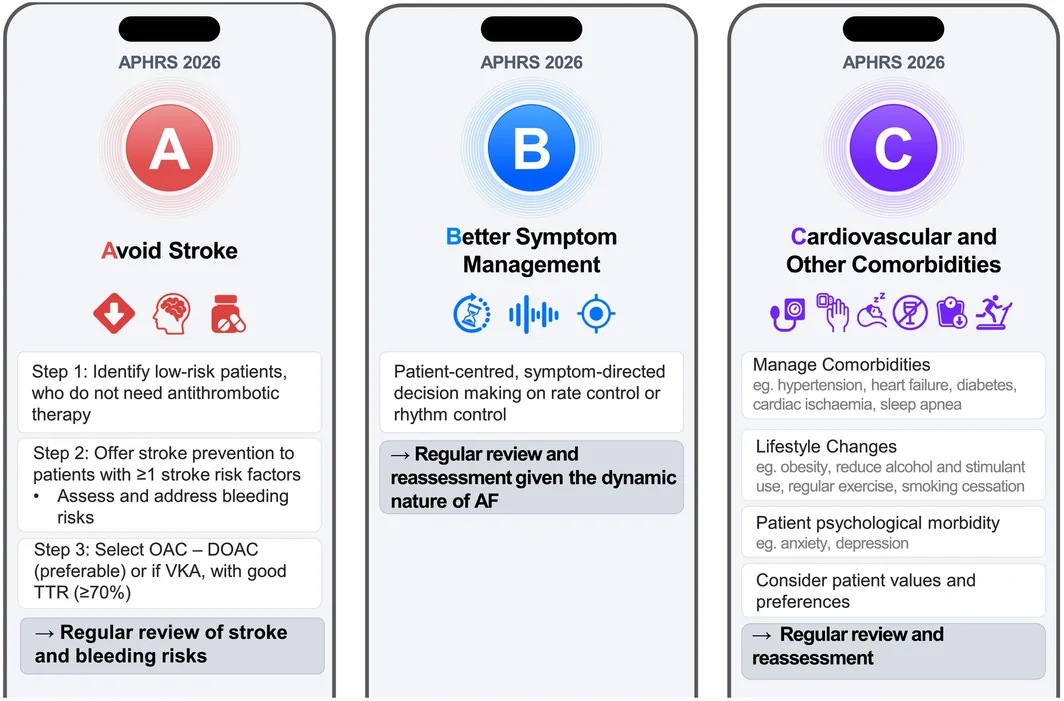
ABC pathway for integrated care management (adapted from Lip et al. [92]). DOAC, direct oral anticoagulant; OAC, oral anticoagulant; TTR, time in therapeutic range; VKA, vitamin K antagonist.

#### 3.1.1 “A” Avoid Stroke

This involves a three‐step approach:

- Identify low‐risk patients (i.e., stroke or thromboembolism event rate < 1% per year) who do not require therapy.
- Offer stroke prevention to those who have one or more risk factors for stroke or thromboembolism.
- Select an appropriate oral anticoagulant—either a direct oral anticoagulant or a vitamin K antagonist with well‐managed time in therapeutic range—while simultaneously assessing and addressing modifiable bleeding risks

#### 3.1.2 “B” Better Symptoms Management

This step emphasizes patient‐centered, symptom‐guided decisions concerning rate versus rhythm control. While traditionally focused on symptom relief and functional status, contemporary evidence supports the benefits of rhythm control in improving cardiovascular outcomes.

#### 3.1.3 “C” Cardiovascular and Other Comorbidities

This addresses concurrent risk factors, manages comorbidities such as hypertension and heart failure, and encourages lifestyle changes, including reducing obesity and engaging in exercise. In addition, attention to patient psychological morbidity as well patient values and preferences is needed.

In all components of A, B and C, regular review and reevaluation is recommended, as with all cardiovascular conditions, since risks (stroke, bleeding, etc.) are not static but dynamic in nature, and decisions on rate or rhythm may change given the dynamic nature of the arrhythmia. Comorbidities and lifestyle may change over time, with aging and incident risk factors. The ABC pathway provides a framework to optimize guideline‐adherence of evidence‐based care based on the A, B and C components.

The efficacy of the AF Better Care (ABC) pathway is well established across Asian populations. The cluster‐randomized mAFA‐II trial in China [93] highlighted its value, showing that mobile‐facilitated ABC care improved guideline adherence and reduced the composite outcome of ischemic stroke/thromboembolism, death, and AF‐related hospitalization by 61%. Of note, baseline proportions of OAC use in the usual care and mAFA App (implementing ABC pathway) arms were 48.4% and 66.1%, respectively. At follow‐up, the proportions of high‐risk patients (CHA_2_DS_2_‐VASc ≥ 2 in males, ≥ 3 in females) receiving OAC were 90.5% in the mAFA App arm, versus a decline in the usual care arm (45.3%).

Ancillary analyses from the mAFA‐II trial show that the ABC pathway intervention showed consistency of benefit irrespective of age [94] and sex [95] as well as high‐risk subgroups with multimorbidity [96], heart failure [97], prior stroke [98], diabetes [99] and clinically complex phenotypes [100]. The long‐term extension cohort showed high adherence and persistence with the mAFA intervention [39]. Based on the mAFA trial data, a health economic analysis demonstrated the cost‐effective use of mHealth apps in streamlining and integrating care via the ABC pathway for AF [101].

The MIRACLE‐AF trial was a cluster randomized trial whereby the ABC pathway was delivered by village doctors in rural China supported by telehealth, and was compared to usual care [102]. There was improvement in guideline adherence and the composite cardiovascular event outcome was significantly lower in the intervention group compared to the usual care group (6.2% versus 9.6% per year; HR 0.64; 95% CI, 0.50–0.82). Baseline proportions of OAC use in the *intervention* and *usual care* arms were 10.9% and 10.7%, respectively, and at 12 months, guideline adherent OAC use increased to 85.2% and 20.8%, respectively.

Prespecified subgroup analyses show consistency of the ABC pathway intervention in the MIRACLE‐AF trial for the elderly [103], and irrespective of cognitive function strata [104]. Also, this telemedicine‐based, village doctor‐led integrated AF management was a potential cost‐effective strategy in resource‐limited rural settings [105].

In Europe, the AFFIRMO trial (NCT06775028) investigated the efficacy of the ABC pathway in improving guideline adherence together with the comprehensive geriatric assessment (CGA) in elderly AF patients with multimorbidity in a cluster randomized trial [106]. The AFFIRMO trial did not show any significant difference between the intervention arm versus usual care in a trial population with low event rates that was very well treated and guideline‐adherent (97% of the enrolled patients with anticoagulated baseline), who were also clinically robust (hence the limited effect of the CGA) [107].

With regards to Asian‐based evidence for this guideline, a pooled individual patient‐level data analysis of the mAFA‐II and MIRACLE‐AF trials showed that the intervention group demonstrated a significantly lower risk of the primary endpoint (a composite of all‐cause mortality, ischemic stroke, hemorrhagic stroke, heart failure (HF), acute coronary syndrome (ACS), and major bleeding events) compared to the control group (adjusted hazard ratio [aHR]: 0.72; 95% CI 0.56–0.93) [108]. No significant heterogeneity was observed between studies, with both trials showing directionally consistent effects favoring the ABC pathway intervention.

The clinical trial results from Asia are well supported by real‐world data from Korea's NHIS Database (*n* = 204 842), Thailand's Cool‐AF registry (*n* = 3402), the Middle East's Gulf SAFE registry (*n* = 2043), and the region‐wide APHRS AF Registry (*n* = 4013) [109, 110, 111, 112]. All of these registries consistently reported that ABC compliance significantly reduces cardiovascular adverse outcomes and all‐cause mortality. However, adherence to the ABC pathway remains suboptimal in patients with AF, but is associated with substantial beneficial effects on prognosis (Figure 6) [113].

**FIGURE 6:**
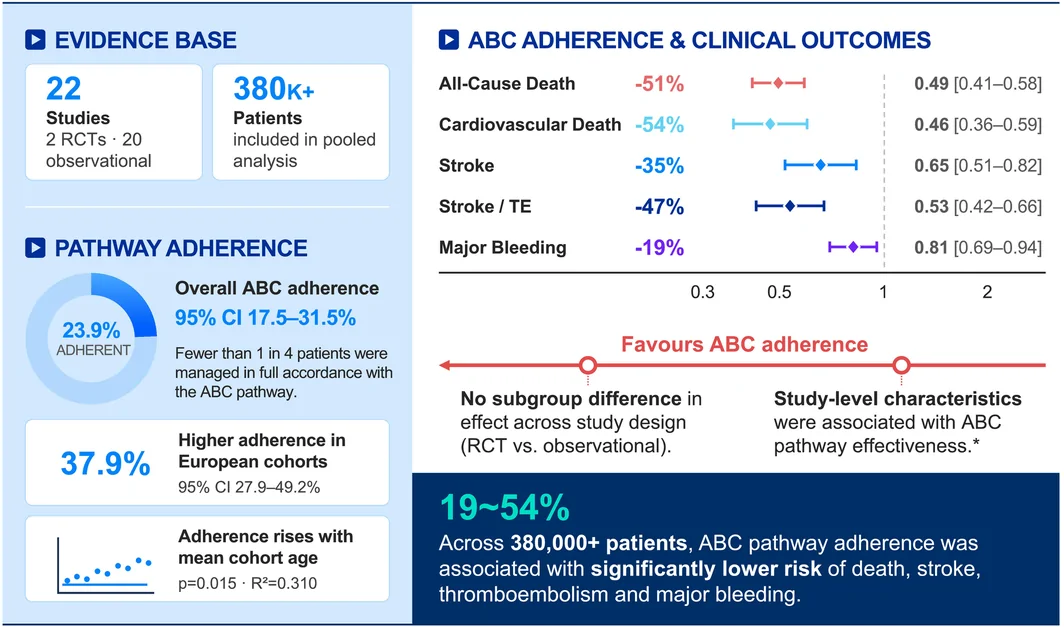
Recent update of “AF better care” pathway (adapted from Romiti et al. [113]). ABC pathway adherence was associated with a lower risk of all‐cause death, cardiovascular death, stroke, stroke/TE, and major bleeding. *Study level characteristics, include geographical location, mean age, prevalence of comorbidities, and potential confounders.

**Table:** 

Recommendations	Class	Level
Integrated patient‐centered management of AF, organized according to the evidence‐based “ABC” pathway (Avoid stroke, Better symptom management, Cardiovascular and comorbidity optimization), is recommended to reduce mortality, stroke, and AF‐related hospitalizations [92, 93, 109, 110, 111, 112]	I	A

### 3.2 Integrated AF Care in Asia

The Asia‐Pacific region is a vast territory characterized by substantial heterogeneity in healthcare systems, resource availability, cultural practices, and languages. Consequently, while the principles of integrated care are universal, their implementation requires adaptation to local contexts to ensure patient understanding and compliance. One example of bridging cultural and linguistic barriers is the Thai guideline, which adapted the ABC pathway into the “No 3 Aw” principle [114]. This mnemonic translates the pathway into three distinct goals, each beginning with the Thai letter “Aw”: no stroke; no symptoms; no obesity, and others. Such culturally adapted approaches may facilitate patient engagement, improve understanding, and enhance adherence to integrated AF care strategies.

In East Asia, comprehensive healthcare systems and large‐scale healthcare databases have enabled evaluation of integrated AF care in routine clinical practice. In South Korea, nationwide cohort studies based on the Korean National Health Insurance system have demonstrated that adherence to integrated AF care using the ABC pathway is associated with significantly improved clinical outcomes, including reduced risks of stroke, mortality, and hospitalization [109, 115, 116]. In China, both real‐world registry data and randomized clinical trial evidence support integrated AF care. Data from the ChiOTEAF registry demonstrated improved clinical outcomes associated with ABC pathway adherence [117], and as discussed above, the mAFA‐II and MIRACLE‐AF trials further demonstrated that mobile health‐implemented integrated AF care significantly improved clinical outcomes in patients with AF [93, 108, 118]. These findings provide strong evidence supporting the clinical benefit and feasibility of integrated AF care in East Asian populations.

In other parts of Asia, regional registry studies provide important evidence supporting integrated AF care. In Southeast Asia, the COOL‐AF registry in Thailand demonstrated improved clinical outcomes associated with adherence to the ABC pathway [110]. In South Asia, the KERALA‐AF registry in India demonstrated the feasibility of implementing holistic integrated AF care and characterized clinical phenotypes and management patterns in patients with AF [119]. In the Middle East, the Gulf SAFE registry demonstrated that adherence to integrated AF care was associated with improved clinical outcomes [111]. Collectively, these regional registry studies provide consistent real‐world evidence supporting the implementation of integrated AF care across diverse healthcare systems in Asia.

In addition to region‐specific studies, multinational registry data provide important evidence supporting integrated AF care across Asia. The APHRS AF registry, which enrolled patients from five Asian countries (Hong Kong, South Korea, Japan, Singapore, and Taiwan), demonstrated that adherence to integrated AF care using the ABC pathway was associated with significantly improved clinical outcomes across diverse healthcare systems and clinical settings (Figure 7). These multinational findings provide robust evidence supporting the generalizability and clinical effectiveness of integrated AF care throughout Asian populations [107, 108, 112, 120].

**FIGURE 7:**
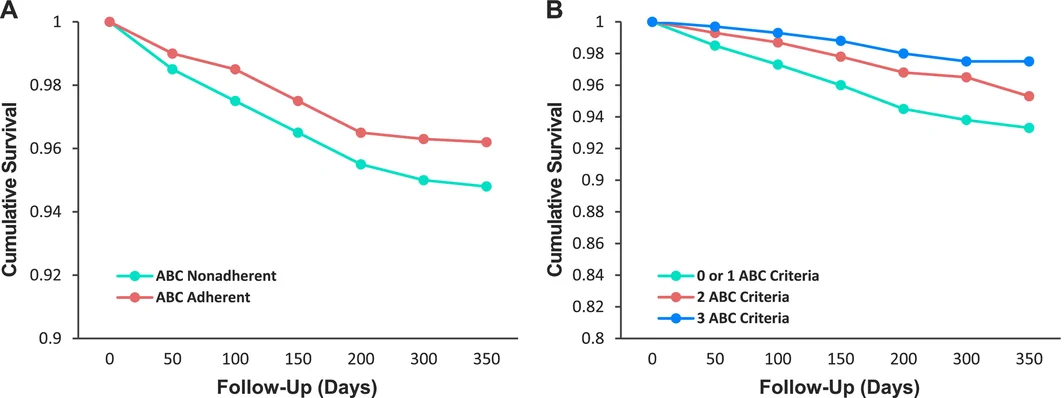
Association between ABC pathway adherence and clinical outcomes in the multinational APHRS AF Registry (adapted from Bucci T, et al. *JACC: Asia*. 2023; 3 (4): 580–591) [112]. (A) Risk of composite outcome in ABC‐adherent versus ABC‐nonadherent patients. (B) Risk of composite outcome according to the number of fulfilled ABC criteria (0–1 vs. 2–3 criteria).

Despite increasing evidence supporting the effectiveness of integrated AF care in Asia, substantial regional variation remains in its implementation, and real‐world adherence remains suboptimal in many countries. Barriers include differences in healthcare infrastructure, resource availability, and provider and patient awareness. Digital health technologies, including mobile health platforms, may facilitate implementation and improve adherence to integrated AF care. Clinical trial evidence from Asia, particularly the mAFA‐II trial, supports the role of mobile health–based integrated management as an effective strategy to enhance implementation and improve clinical outcomes [93]. Continued efforts are needed to promote broader adoption and implementation of integrated AF care across Asia to optimize outcomes for patients with AF.

## 4 Healthcare Burden of AF in Asian Countries

### 4.1 East Asia, Southeast Asia, South Asia

AF imposes a substantial burden across Asia [121], associated with increased risks of mortality, stroke, bleeding complications, and healthcare utilization. The magnitude and drivers of this burden vary across regions, reflecting differences in healthcare systems, socioeconomic conditions, access to therapies, and population aging.

In East Asia, including China, Japan, South Korea, and Taiwan, AF imposes a substantial and increasing healthcare burden, driven by rising prevalence and rapid population aging. These countries have some of the most rapidly aging populations globally, resulting in a marked increase in the number of elderly individuals at high risk for AF and its complications [3, 10, 14, 122].

This demographic shift in East Asia has led to marked increases in AF‐related healthcare utilization, including hospitalizations, outpatient visits, and long‐term care needs [3, 10]. For example, in Korea, AF‐related hospitalizations and healthcare costs increased by 4.2‐fold and 5.7‐fold, respectively, over the past decade [34]. In addition, improved survival from cardiovascular diseases and increased detection of AF have contributed to a growing population requiring long‐term management. As a result, despite well‐developed healthcare systems and broad access to evidence‐based therapies, the burden of AF in East Asia continues to rise, primarily driven by population aging and increased longevity.

In Southeast Asia and South Asia, AF is associated with a particularly high clinical and healthcare burden. Mortality rates in these regions are relatively high compared with global and other regional data in Asia. For example, the Kerala‐AF registry from India reported a mortality rate of 16.5%, markedly higher than the 2.7% observed in the APHRS‐AF registry, which included East Asian populations and Singapore [123, 124]. AF‐related complications, including stroke and bleeding, contribute significantly to adverse clinical outcomes and healthcare utilization across these regions.

Economic and infrastructure barriers represent major contributors to the healthcare burden. Economic barriers significantly restrict access to guideline‐approved therapies, particularly DOACs, and vitamin K antagonists (VKAs) remain the most commonly used oral anticoagulants in many countries within Southeast Asia and South Asia [123, 125]. Although the increasing availability of generic DOACs is reducing cost disparities and facilitating broader adoption, access to these therapies remains uneven, particularly in lower‐resource settings. Moreover, effective VKA management remains inadequate in these regions, largely due to limited healthcare personnel and low patient awareness [123, 126, 127]. In the Kerala‐AF registry, nearly half of patients receiving VKA therapy were unaware of their INR status [123]. Furthermore, logistical barriers are especially pronounced in rural regions. For example, in Chhattisgarh, India, patients were required to travel approximately 78 km to access healthcare facilities, and anticoagulation control remained poor, with median time in therapeutic range (TTR) as low as 13% [128].

In East Asia, population aging and increased longevity are the primary drivers of the increasing healthcare burden. In contrast, in Southeast Asia and South Asia, economic constraints, limited access to care, and infrastructure limitations are major contributors. Addressing both demographic and healthcare system factors will be essential to reduce the healthcare burden of AF across Asia.

### 4.2 Healthcare Burden of AF in Western and Central Asia

AF continues to impose a major morbidity and mortality burden in Central Asia, and North Africa and the Middle East Region (MENA) [129]. According to the Global Burden of Disease 2023, age‐standardized rate of AF was 662 and 439 per 100 000 in Central Asia and the MENA, respectively [129]. AF contributed to 1648 deaths and 66 285 disability adjusted life‐years in Central Asia, and 12 916 deaths and 324 272 disability‐adjusted life‐years in MENA in 2023 [129].

Patients in the MENA region presenting with AF are reported to be younger than their Western counterparts [130, 131]. Gulf SAFE (*n* = 2043), a registry of patients with AF presenting to the emergency department, documented suboptimal anticoagulation use [129]. In the non‐valvular AF cohort of the GULF SAFE (*n* = 1721), 1‐year stroke/transient ischemic attack was 4.2%, while all‐cause mortality was 15.3% [132]. Mean total annual costs per patient attributable to AF were $1151 per person in the UAE and $3001 per person in Saudi Arabia, with monitoring costs being the largest contributor to costs in both countries [133]. In the GARFIELD‐AF Study Middle East (*n* = 1660 from Egypt, UAE, Turkey) of patients with newly diagnosed non‐valvular AF, one‐year stroke and bleeding rates were lower, although mortality was relatively higher compared to the non‐Middle East cohort [134]. Although the Middle East cohort had increased antithrombotic use, appropriate DOAC use was lower (57.1% vs. 74.4% globally) [134]. The FLOW‐AF registry (*n* = 1418 from Egypt, Lebanon, Saudi Arabia, UAE) documented high DOAC adoption (65.8%) with low one‐year event rates: bleeding events (1.7%), transient ischemic events (1.7%), all‐cause mortality (1.7%), stroke (0.6%), myocardial infarction (0.2%), and systemic embolism (0.08%) [131]. The UAE non‐valvular AF cohort was characterized by a younger demographic and lower risk scores relative to global counterparts [133]. Collectively, these data suggest improved anticoagulation uptake and lower short‐term event rates in contemporary MENA cohorts over the past decade. Central Asian countries lack comparable large prospective AF registries, and data on anticoagulation uptake and quality of care remain limited.

## 5 Healthcare Systems—Urban Versus Rural

### 5.1 Urban Healthcare System of AF

Urban healthcare systems in countries such as Japan, South Korea, Taiwan, Singapore, and major metropolitan areas of China are characterized by dense hospital networks, strong specialist availability, comprehensive insurance coverage, and high adoption of guideline‐directed AF care. These high‐income East Asian settings have experienced rapid population aging, resulting in a growing AF burden managed largely within tertiary and secondary urban centers [135].

National and insurance‐based databases from East Asia demonstrate increasing AF prevalence and improved detection in metropolitan areas, supported by widespread access to electrocardiography, ambulatory monitoring, and specialist cardiology services [136]. In Korea, nationwide analyses show socioeconomic and regional variation in AF prevalence and healthcare utilization, but urban residents generally have more consistent hospital follow‐up and specialist access [137, 138]. Similarly, Taiwan's universal National Health Insurance system facilitates broad access to anticoagulation and rhythm management in urban hospitals, contributing to high rates of OAC use in eligible patients.

Stroke prevention strategies in urban East Asia have shifted substantially toward DOACs, following pivotal trials demonstrating superiority or non‐inferiority versus VKA [139, 140]. Contemporary Asia‐Pacific and international guidelines recommend DOACs as first‐line therapy for most patients with non‐valvular AF [64, 141, 142, 143]. Urban centers in Japan, Korea, and Taiwan have rapidly implemented DOAC‐based strategies, supported by reimbursement systems and specialist familiarity. This transition has improved safety profiles compared with historical VKA management, which required careful TTR monitoring—often suboptimal in real‐world Asian cohorts [144].

Beyond anticoagulation, urban AF care increasingly incorporates integrated management aligned with the ABC pathway, emphasizing stroke prevention, symptom control, and comorbidity optimization [92]. Multicenter Asia‐Pacific registry data demonstrate that adherence to the ABC pathway is associated with lower risks of mortality, stroke, and hospitalization [89, 112, 145]. Urban tertiary hospitals are more likely to provide multidisciplinary AF clinics, electrophysiology services, catheter ablation programs, and structured follow‐up systems addressing multimorbidity—an important issue in aging East Asian populations [112, 146].

Nevertheless, challenges remain. Even in well‐resourced urban systems, residual adverse events persist despite anticoagulation [147], and non‐cardiovascular comorbidities contribute significantly to mortality [148]. Health system pressures from aging demographics, increasing AF prevalence, and rising healthcare costs require continued emphasis on digital health, structured risk stratification, and prevention strategies [135, 149].

### 5.2 Rural Healthcare System

In rural health systems, AF is commonly characterized by a triad of interrelated challenges: delayed or under‐recognition due to constrained diagnostic capacity, undertreatment arising from limited delivery of guideline‐directed and integrated care, and a disproportionately high complication burden, manifested by recurrent hospitalizations and greater downstream disability that intensify pressure on already resource‐limited services.

A consistent profile of suboptimal AF management further distinguishes rural Asia, where therapeutic options are limited and implementation of guideline‐directed care is substantially lower than in urban centers. Large regional AF registries show reduced use of oral anticoagulation and inconsistent application of rate‐ and rhythm‐control strategies in non‐urban settings. VKA remains the predominant anticoagulant in many rural hospitals despite clear evidence that it is inferior to DOAC in both efficacy and safety, especially among Asian populations with higher risks of intracranial hemorrhage. Maintaining stable VKA control is further hindered by irregular INR monitoring, with the COOL‐AF registry showing time‐in‐therapeutic‐range values below recommended thresholds in rural Thai practice [150]. Longitudinal data from China similarly demonstrate that many patients remain outside anticoagulation targets despite ongoing healthcare contact [144]. Implementation of integrated AF pathways is also limited: adherence to the ABC pathway in rural Chinese cohorts remains extremely low (ranging from 5% to 10%), reflecting the difficulty of operationalizing multi‐component care in resource‐constrained environments [151, 152].

The epidemiological consequences of these disparities are reflected in patterns of hospitalization and health‐system demand. Longitudinal AF registry data indicate frequent recurrent admissions over relatively short follow‐up periods, particularly among patients with multiple comorbidities, underscoring the cumulative burden AF imposes on healthcare facilities—burden that is amplified in rural settings with limited bed capacity and specialist support [62]. Broader analyses of AF care across Asia note that recurrent hospitalizations for heart failure, bleeding, or stroke carry substantial financial implications for rural households [62, 153], which often rely on out‐of‐pocket payments and lack formal financial protection. Data from Vietnam stroke registry including 94 144 patients showed that up to 15% of the patients had undiagnosed AF [154]. Commentaries focused on South and Southeast Asia further emphasized that AF‐related stroke and disability exert disproportionate long‐term impact in rural areas, where access to acute stroke care, rehabilitation, and long‐term support services is limited [155].

The diagnosis and management of AF in rural Asia are therefore characterized by reduced opportunities for timely detection, persistently suboptimal delivery of evidence‐based therapy, and a heightened vulnerability to AF‐related complications, reflecting the structural constraints embedded within rural health systems.

### 5.3 Healthcare System—Urban Versus Rural: Key Disparities and Challenges in the Asia‐Pacific Region

Across the Asia‐Pacific region, substantial urban–rural disparities exist in the healthcare delivery of AF. Urban centers are characterized by dense hospital networks, specialist cardiology services, access to electrophysiology laboratories, and higher uptake of DOACs in accordance with regional and international guidelines [64, 142, 143]. These systems increasingly adopt integrated, multidisciplinary models aligned with the ABC pathway, which has been associated with improved clinical outcomes [89, 92, 112, 145].

In contrast, rural areas face structural limitations. These include shortages of cardiologists, limited diagnostic infrastructure (e.g., Holter monitoring, echocardiography), and reduced access to DOACs due to cost or formulary constraints. VKA remains widely used, yet maintaining optimal TTR can be challenging where INR monitoring is inconsistent [144]. Lower awareness, delayed diagnosis, and fragmented follow‐up further contribute to suboptimal stroke prevention and higher complication rates [155, 156, 157].

Additionally, the aging population and high burden of multimorbidity amplify system strain across both settings [135, 146]. Addressing disparities requires scalable screening strategies, telemedicine‐supported shared care, workforce strengthening, and policies ensuring equitable access to guideline‐directed anticoagulation throughout the region.

Recommendations: AF Healthcare System (Urban–Rural Disparities, Asia‐Pacific).

**Table:** 

Recommendation	Class	Level
Healthcare systems should ensure equitable access to guideline‐directed AF diagnosis and management across urban and rural populations, addressing disparities in awareness, access to care, and treatment observed in Asia‐Pacific regions [137, 138, 155, 156, 158]	I	C
Systematic AF screening and early detection strategies (e.g., opportunistic pulse check or single‐lead ECG in primary care or community programs) should be implemented equitably across urban and rural populations, particularly among older individuals and those with limited access to healthcare, to improve early diagnosis and reduce stroke risk [149, 157, 159]	I	A
Oral anticoagulation should not be withheld due to geographic location, and guideline‐directed stroke prevention therapy should be implemented equally in rural and urban settings, as anticoagulation significantly reduces stroke risk in AF [64, 90, 139, 140, 142, 160]	I	A
Integrated AF care based on the ABC pathway should be implemented across healthcare systems, including rural care networks, as adherence to this strategy improves major clinical outcomes [89, 92, 112, 145, 151, 152]	I	B
Telemedicine and hub‐and‐spoke referral systems linking rural providers with urban specialist centers should be considered to improve access to AF expertise, anticoagulation management, and comprehensive care [161, 162, 163, 164]	IIa	B

## Funding

The authors have nothing to report.

## Ethics Statement

This article is a guideline document and does not involve human participants or the collection of individual‐level data.

## Conflicts of Interest

Boyoung Joung has served as a speaker for Bayer, BMS/Pfizer, Medtronic, and Daiichi‐Sankyo and has received research funding from Samjin, Yuhan, Medtronic, Boston Scientific, and Abbott Korea. Rungroj Krittayaphong reports speaking fees from Boehringer Ingelheim, Bayer, Pfizer, and Daiichi‐Sankyo. Eue‐Keun Choi reports receiving research grants or speaking fees from Abbott, Bayer, BMS/Pfizer, Biosense Webster, Chong Kun Dang, Daewoong Pharmaceutical Co., Daiichi‐Sankyo, DeepQure, Jeil Pharmaceutical Co. Ltd., Medtronic, Samjinpharm, Samsung Electronics Co. Ltd., Seers Technology, Skylabs, and Yuhan Corporation. Hui‐Nam Pak reports receiving honoraria from Abbott, Medtronic, and Boston Scientific Inc. and research grants from Medtronic and Abbott Inc. Tze‐Fan Chao reports speaking fees from Abbott, Boehringer Ingelheim, Bayer, Pfizer, Daiichi‐Sankyo, Biotronik, and Medtronic. Gregory Y.H. Lip reports institutional consultancy, advisory, and/or speaker roles for BMS/Pfizer, Boehringer Ingelheim, Anthos, Boston Scientific, and Huawei; no fees are received personally. All other authors report nothing to disclose.

## Data Availability

The data that support the findings of this study are available from the corresponding author upon reasonable request.
